# Copine 7 promotes colorectal cancer proliferation through PKM2 interaction and MAPK signaling pathway

**DOI:** 10.3389/fonc.2023.1166444

**Published:** 2023-07-04

**Authors:** Tianwen Yu, Changhao Huang, Chen Lai, Qing He, Weijie Yuan, Zihua Chen

**Affiliations:** ^1^ Department of Gastrointestinal Surgery, Xiangya Hospital, Central South University, Changsha, China; ^2^ The Hunan Provincial Key Lab of Precision Diagnosis and Treatment for Gastrointestinal Tumor, Xiangya Hospital, Central South University, Changsha, China; ^3^ International Joint Research Center of Minimally Invasive Endoscopic Technology Equipment and Standardization, Xiangya Hospital, Central South University, Changsha, China; ^4^ Department of Organ Transplantation Center, Xiangya Hospital, Central South University, Changsha, China

**Keywords:** colorectal cancer, CPNE7, PKM2, MAPK pathways, proliferation

## Abstract

**Introduction:**

Colorectal cancer (CRC) is currently the third most common cancer in the world, and its prevalence and mortality rate continue to increase.

**Methods:**

Based on an analysis of The Cancer Genome Atlas database, Tumor Immune Estimation Resource and Gene Expression Profiling Interactive Analysis, we explored the expression of CPNE7 in tumors. Immunohistochemistry and quantitative polymerase chain reaction analysis the expression of CPNE7 in colorectal cancer. Our study explored how CPNE7 promotes CRC cell proliferation and migration in vitro and in vivo. Transcriptome sequencing and Co-IP assay explored the underlying mechinaism of CPNE7 founction.

**Results:**

We found the CPNE7 was overexpressed in CRC by database and IHC. CPNE7 promoted CRC cells proliferstion and migration in vitro and in vivo. Comparing and analyzing transcriptome sequencing between exogenous up-/downregulated CPNE7 CRC cells and the controls, we found that CPNE7 activates mitogen-activated protein kinase (MAPK) signaling pathway stimulating cancer cell proliferation. Coimmunoprecipitation experiments revealed an interaction between CPNE7 and pyruvate kinase muscle protein (PKM2). We also found the activity of MAPK signaling is regulated by exogenous CPNE7 expression.

**Discussion:**

These results imply that CPNE7 may promote the progression of CRC by interacting with PKM2 and initiating the MAPK signaling pathway.

## 1 Introduction

Colorectal cancer (CRC) is the third most common cancer worldwide, ranked third in incidence rate and second in mortality rate among all cancers (1). Normally, CRC begins with an aberrant crypt, evolving into a neoplastic precursor lesion, and eventual progressing to cancer (2). Inactivation of tumor suppressor genes and activation of oncogenes are vital accelerators in the progress of cancer (3). CRC usually is asymptomatic until it develops to an advanced stage. Although treatments such as surgery and chemoradiotherapy have improved, the age-standardized 5-year relative survival rate has not significantly improved (4, 5). Therefore, the underlying molecular mechanism of CRC progression needs to be uncovered.

Copines (CPNEs), a novel group of super proteins numbered between 1 and 8, are calcium-dependent phospholipid binding proteins, that were discovered after being isolated from extracts of *Paramecium tetraurelia* (6). While CPNEs are expressed throughout various mammal tissues, CPNE6 is expressed specifically in neural tissue (6, 7). The protein families of CPNEs are composed of two C2 domains in the N-terminal and an A-domain in the C-terminal. The two C2 domains (C2A, C2B) are key structures regulating calcium and phospholipids (7). The C2B domain could assist CPNEs to locate cell membranes in a calcium-dependent, phospholipid-binding manner (8, 9). Additionally, C2 domains are known to increase the phosphorylation of extracellular regulated protein kinase (ERK) (10). CPNEs appear to have different functions in various tumors (11–13). One study found that CPNE1 decreases NF-kB transcription *via* interaction with p65 and promotes p65 endoproteolysis for regulating the development of cancer (14). CPNE3 has been found to contribute to breast cancer motility based on interaction with ErbB (phosphorylated Tyr1248 of ErbB2) (15). However, the role of CPNEs in CRC oncogenesis and progression remains obscure.

Serine/threonine kinase, known as Akt, is involved in cell survival and migration, glucose metabolism, and protein synthesis (16–18). It has been observed widely in biomedical research and is known as an intermediary, facilitating communication between other protein-regulating pathways. For example, PIP3 and PDK1 together phosphorylate Akt, then later regulate cell growth by intersecting with TSC1/2 compound and the mTOR signal pathway (19–21). Activation of the Akt signaling pathway has been reported in more than 40% of cancers, including breast and colorectal cancers. Mitogen-activated protein kinase (MAKP) is another signaling pathway in which extracellular regulated protein kinases (ERK) play an important role. The phosphorylated active form of ERK, known as p-ERK1/2, relocates from cytoplasm into nucleus to regulate oncogenic transcription factors such as c-Jun (22, 23). The ERK pathway is activated in many tumor types, and contributes to such functions as neoplasm cell proliferation, metastasis, invasion, and drug resistance (24, 25). SYT14L, which has the same C2 domain as CPNE7, plays an important role in melanocyte differentiation by increasing the phosphorylation of ERK. CPNE1, a type of CPNE, can enhance CRC cell growth and drug resistance *via* the AKT-GLUT1/HK2 pathway (26). The PI3K-Akt and MAPK signaling pathways are two main mechanisms contributing to cancer cell proliferation, survival, differentiation, and motility (27). Therefore, we hypothesized that CPNE7 could regulate CRC proliferation through the MAPK signal pathway.

## 2 Materials and methods

### 2.1 Immunohistochemical staining

We collected tumor samples surgically removed from 75 CRC patients diagnosed between April 2004 and January 2013. The 75 patients, including 38 male subjects and 37 female subjects, ranged in age from 33 to 86 and received neither radiotherapy nor chemotherapy before surgery.

We formed a tissue microarray chip with the samples, then performed IHC staining for CPNE7 (1:300) on the chip. After staining, whole slides were screened into the Slideviewer System. Results were evaluated simultaneously by two independent pathologists to obtain the percentage of tumor stained and its corresponding score (1: < 25%, 2: 25–50%, and 3: > 50%) as well as staining intensity (1: negative, 2: weak, 3: moderate, and 4: strong). The percentage score multiplied by staining intensity was represented as the final score, with a score greater than 6 considered to be a significant overexpression. This research was approved by the Ethics committee of Xiangya Hospital Central South University.

### 2.2 Total RNA isolation and polymerase chain reaction analysis

We scissored the tumor samples into fragments of appropriate sizes (or the appropriate number of cells), placed them in the Trizol reagent (Takara Bio, Dalian, China), and extracted the total RNA after grinding. We then used a reverse transcription kit from Takara Bio to reverse transcribe the total RNA into complementary DNA. Real-time PCR was performed using PCR mix (Vazyme, Nanjing, China) in Bio-Rad CFX Maestro (Hercules, USA).

For CPNE7, the forward primer (5’→3’) was 5’-TGCTCAAGTTTGGCAGGAAC-3’; and reverse primer was 5’-GCTCCACGTAGCCGTTGTT-3’. For Glyceraldehyde-3-phosphate dehydrogenase (GAPDH) the forward primer was 5’-CGACCACTTTGTCAAGCTCA-3’ and reverse primer was 5’-CCCTGTTGCTGTAGCCAAAT-3’.

### 2.3 Western blot

Cells were lysed in RIPA (Beyotime, Shanghai, China) and quantified by bicinchoninic acid protein assay (NCM, Suzhou, China). Lysates were separated by SDS-PAGE gel and transferred to Millipore membrane (Millipore, Billerica, USA) using Tris-Glycine solution. To block nonspecific binding, we used 5% fat-free milk, 1* tri-buffered saline solution, and 0.1% Tween-20 solution for 1 hour at room temperature. The membrane was incubated in the primary antibodies overnight at 4°C. The membranes were incubated with secondary antibody at room temperature for 1 hour; then, the chemiluminescence signal was detected using the ECL kit (Millipore, Billerica, USA). GAPDH was used as the control.

The following primary antibodies were used in this research: anti-CPNE7 (1:1000, Proteintech), (1:300, Invitrogen, USA); anti-GAPDH (1:1000, Servicebio); anti-PKM2 (1:1000, Proteintech); anti-HA (1:1000, CST); anti-FLAG (1:1000, CST); anti-AKT (1:1000, CST); anti-AKT^s473^(1:1000, CST); anti-c-Raf (1:1000, CST); anti-ERK (1:1000, Proteintech); anti-p-ERK (1:1000, Proteintech); anti-rabbit IgG, HRP-linked antibody (1:2000, CST), anti-mouse IgG, and HRP-linked antibody (1:2000, CST).

### 2.4 Cell lines and culture

All cell experiments were performed using sterile tools. The HEK293 cell line and human colorectal carcinoma cell HCT116 and DLD1 cell lines were purchased from Procell Life Science & Technology (Procell, Wuhan, China). All cells were cultured in a Thermo incubator at 37°C in 5% CO2, with each line in a different type of medium. The HCT116 cells were cultured in McCoy’s 5A medium (Procell, Wuhan, China) with 10% fetal bovine serum (FBS) (Procell, Wuhan, China). The DLD1 cells were cultured in RPMI-1640 medium (Procell, Wuhan, China), and Dulbecco’s Modified Eagle Medium (Procell, Wuhan, China) was used for the HEK293 cells.

### 2.5 Proliferation assay

Cell proliferation was measured using 3-(4,5-dimethyl-2-thiazolyl)-2,5-diphenyl-2-H-tetrazolium bromide (MTT) assay. After cell counting, 1,000 cells were placed in each pore of a 96-well plate. At the same time on each of the first, third, and fifth days, MTT was added into the wells and incubated for 4 hours at 37°C. Dimethyl sulfoxide absorbed the formazan, and the absorbed solution was analyzed spectrophotometrically at 570 nm and 630 nm.

### 2.6 Transwell assay

To perform the transwell assay, we used Corning 24-well plates with 8 μm pore size and Matrigel substrate (Corning, New York, USA). We placed between 20,000 and 100,000 cells into the upper chamber, according to the cell invasive ability, and added 10% FBS mediation to the lower chamber. After 24 hours at 37°C in a 5% CO2 basal incubator, cells were fixed with methyl alcohol and stained with 0.1% crystal violet. Cells were counted in four randomly chosen microscopic fields.

### 2.7 Wound healing assay

Cells for the wound healing assay were cultured in the 6-well plates and a 200-μl pipette tip was used to wound the convergent monolayers. We used phosphate-buffered saline to clean the cell debris, then photographed the wound boundary at baseline and 48 hours to record the change. The cell migration rate (%) was calculated as the change in wound distance (0–48 hours) divided by the initial wound distance (0 h).

### 2.8 Colony formation assay

Four hundred stable expression cells were seeded into every well of 6-well plates and incubated at 37°C in a 5% CO2 incubator for 10 days with 10% FBS medium. The colonies were fixed with methyl alcohol and stained with 0.1% crystal violet.

### 2.9 Co-immunoprecipitation assay

Co-IP was performed to detect interaction between proteins. We precleared the cell lysate by incubating it with A/G PLUS agarose at 4°C for 2 hours. Primary antibody was added to the cell lysate and incubated for 1 hour at 4°C in a four-dimensional rotating mixer. Then, 20 μl agarose was added into the mixture and rotated overnight at 4°C. Beads were gathered by centrifugation at 2500 rpm at 4°C. W cold rinsed the beads 4 times, each time repeating the centrifugation step. Beads were boiled in 2× SDS-PAGE loading buffer for 5 minutes. We used Western Blot to analyze the co-IP result.

### 2.10 Animals

We selected 5-week-old nonobese diabetic/severe combined immunodeficiency (NOD/SCID) mice (SLAC animal, Hunan, China) for the cell-derived xenograft model. We cultured stably expressed CPNE7 over-expression (CPNE7-OE) cells and CPNE7 knockdown (CPNE7-KD) cells and control cells. We then subcutaneously injected 5 × 10^6^ cells into the NOD-SCID mice and monitored and measured the xenograft tumors every two days. Tumor volume was measured as (length × width^2^)/2. Animal studies were approved by the Laboratory Animal Welfare Ethics Committee, Central South University.

### 2.11 RNA transcriptome sequencing

RNA was extracted after transient transfected CPNE7-OE and CPNE7-KD lentivirus into the DLD1 and HCT116 cell lines, respectively. Biomarker Technologies (BMK) (Rohnert Park, California, USA) was employed for sequencing analysis, and bioinformation analysis was performed online with the BMKCloud at www.biocloud.net.

### 2.12 Liquid mass spectrometry

We employed Co-IP to explore the possible interactional proteins. The proteins in Co-IP lysate were separated in SDS-PAGE gel. We cut the gel and extracted the proteins and PTM Biolabs company sequenced the proteins.

### 2.13 Statistical analysis

SPSS 26.0 version software and GraphPad Prime 8 were used to statistical analysis. Date was presented as the mean ± SD. Differences between groups were analyzed using two-tailed Students’ *t*-test or unpaired *t*-test. χ^2^ test was employed to analyzed immunohistochemical scored. *p*<0.05 was considered statistically significant.

## 3 Results

### 3.1 Expression and clinicopathology correlation of CPNE7 in CRC tissues

**Figure 1:**
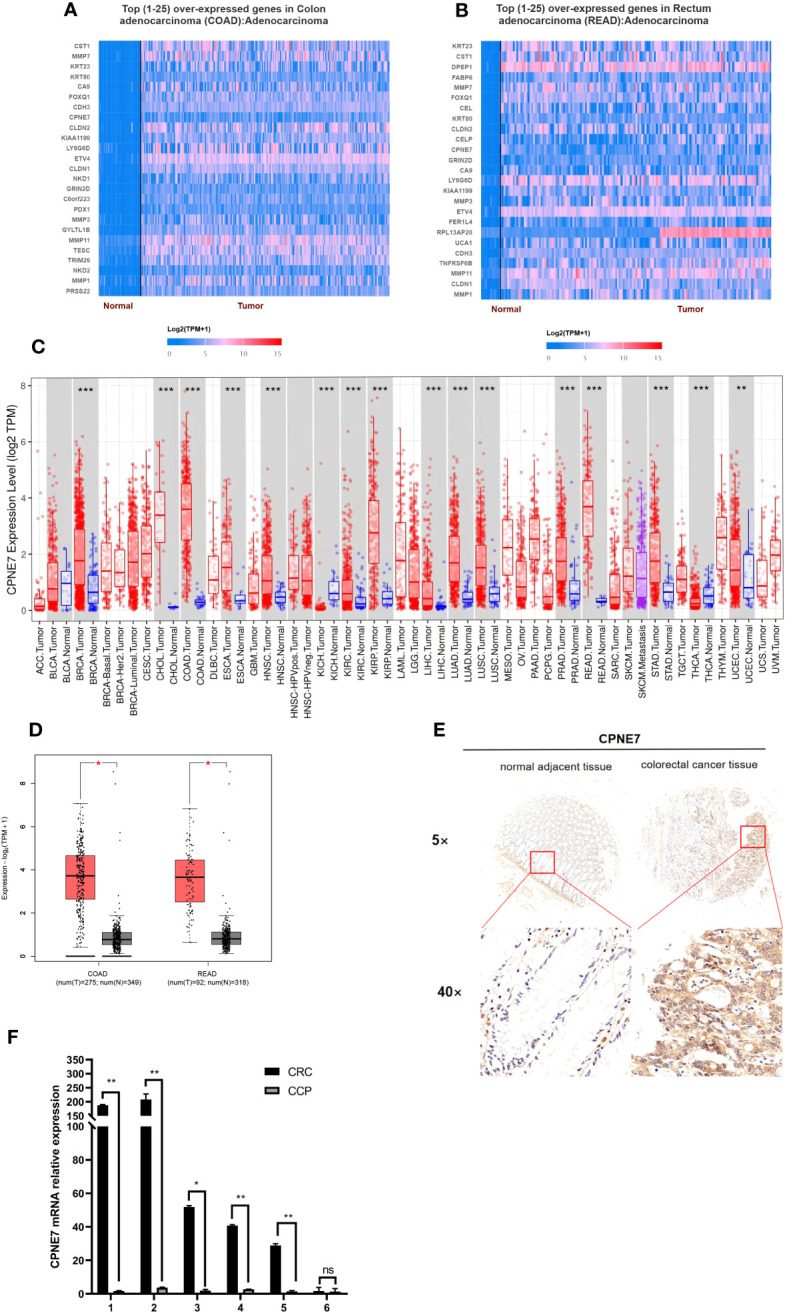
Top 25 over-expressed genes in colon **(A)** and rectum **(B)** adenocarcinoma, from UALCAN online web; **(C)** CPNE7 expression level in different cancer types, from TIMER database (*P*-value Significant Codes: 0 ≤ *** < 0.001 ≤ ** < 0.01 ≤ * < 0.05); **(D)** CPNE7 mRNA expression on box plots in colon and rectum adenocarcinoma, from GEPIA database; **(E)** IHC staining of CRC cells in two patients, compared with adjacent normal tissues; **(F)** CPNE7 mRNA level in six CRC patients by qPCR. *P-*value Significant Codes: 0 ≤** < 0.01 ≤ * < 0.05, ns means nonsignificant).

To identify potential differentially expressed genes (DEGs) in colorectal cancer, we used the University of Alabama Cancer database to access The Cancer Genome Atlas (TCGA) and found that the mRNA level of CPNE7 is highly expressed in CRC tissue compared with adjacent normal tissue (**Figures 1A, B**). The Tumor Immune Estimation Resource revealed that CPNE7 is highly expressed in many tumor types, including colon and rectal adenocarcinoma (**Figure 1C**). Using Gene Expression Profiling Interactive Analysis, we discovered CPNE7 mRNA expression differences (**Figure 1D**), in colon adenocarcinoma and rectal adenocarcinoma.

IHC staining of CRC patient tumor samples further validated that CPNE7 in CRC tissue was significantly higher than that in normal tissue (**Figure 1E**). Using a score based on tumor percentage and staining intensity, we analyzed the correlation between scores and clinicopathological characteristics. We found a significant correlation with high expression of CPNE7 and tumor extension, but not with age, gender, TNM stage, differentiation, distant metastasis, or lymph node involvement (**Table 1**). To further confirm that the mRNA level of CPNE7 in CRC tissue was increased, cDNA was preformed to PCR, compared with adjacent normal tissue (**Figure 1F**).

**Table 1:** Clinicopathological characteristics and CPNE7 expression in CRC tissue.

		Case numbers	CPNE7		*P* value
			Low expression	High expression	
Age (years)					
≤ 50		16	3	13	1.000
> 50		59	11	48	
Gender					
Male		38	9	29	0.258
Female		37	5	32	
Tumor extension (T)					
T1–T2		17	8	9	0.002**
T3–T4		58	6	52	
Lymph node					
N0		41	7	34	0.697
N1–N2		34	7	27	
Metastasis					
M0		64	10	54	0.226
M1		11	4	7	
TNM stage					
I–II		39	6	33	0.188
III–IV		36	8	28	
Differentiation degree					
High-mid differentiation		46	7	39	0.334
Low-undifferentiation		29	7	22	

**p < 0.01, *p < 0.05.

### 3.2 CPNE7 increased cancer proliferation rate and inversion *in vitro*

**Figure 2:**
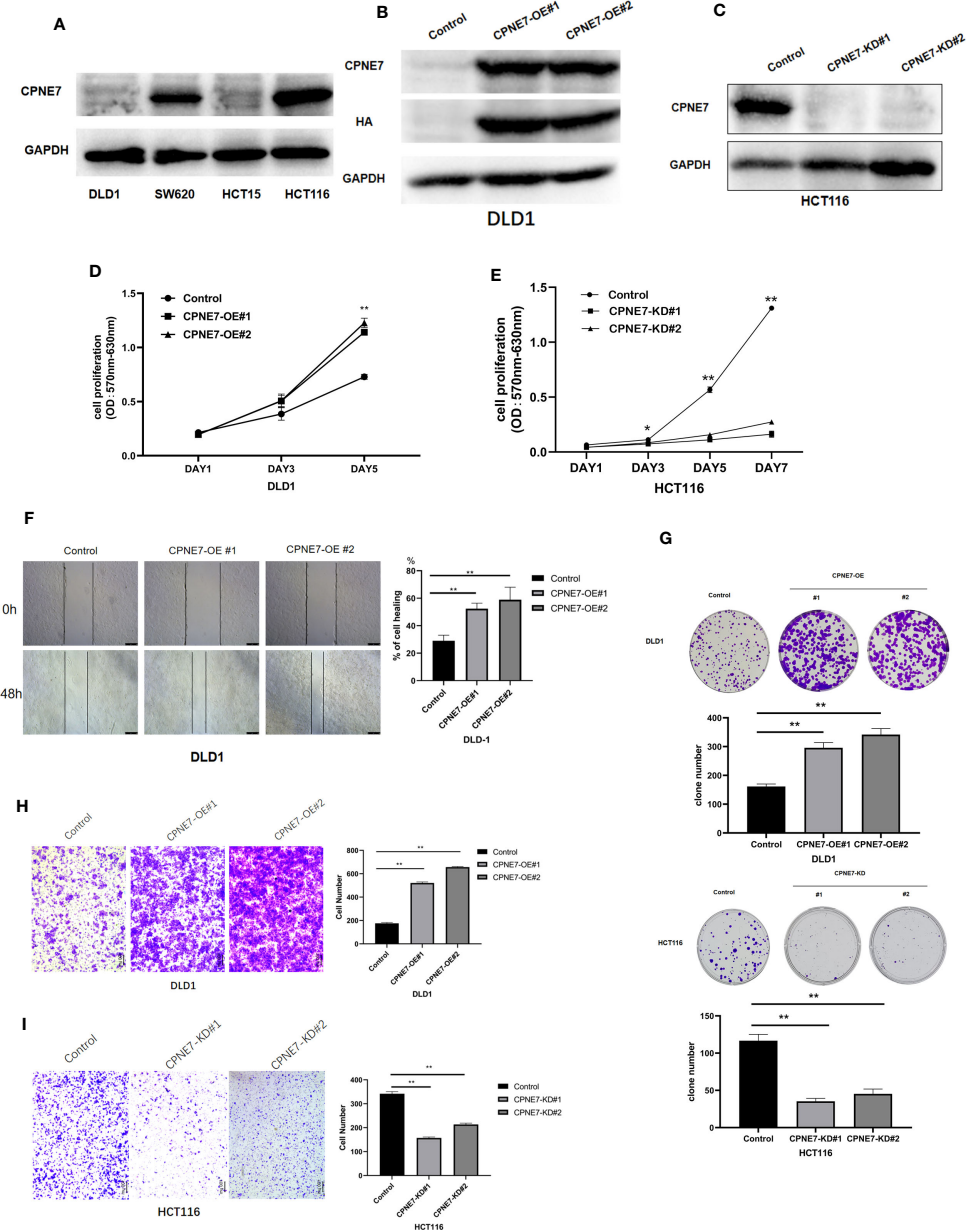
**(A)** Base expression of CPNE7 protein in DLD1, SW620, HCT15, and HCT116 cell lines. Stable expression of CPNE7 protein DLD1 **(B)** and HCT116 **(C)** cells. OE: over-expression; KD: knockdown. Control: a negative control group. **(D, E)** MTT assay of change in cell proliferation by upregulated **(D)** or depleted **(E)** CPNE7, with controls. **(F)** Wound healing assay and healing rate. **(G)** Colony formation assay in DLD1 and HCT116. **(H)** Representative images and quantification data of transwell invasion assay CPNE7-OE and control DLD1 cells. **(I)** representative images and quantification data of transwell invasion assay CPNE7-KD and control HCT116 cells. **p<0.01, *p<0.05.

For the *in vitro* experiment, we first identified the basic CPNE7 protein expression in various wild-type colorectal cancer cell lines, including HCT116, HCT15, SW620, and DLD1. We found that expression of CPNE7 was high in HCT116 cells and low in DLD1 cells (**Figure 2A**). Therefore, to confirm that CPNE7 increases the development of colorectal tumors, we constructed and packaged lentiviruses pLV19-CPNE7 and pLKO.1-shCPNE7. After transfecting the cells with pLV19-CPNE7 lentiviruses, the stably overexpressed CPNE7 (CPNE7-OE) DLD1 was established (**Figures 2B, C**). MTT assay indicated that the CPNE7 facilitated the proliferation of CRC cells (**Figures 2D, E**), and the degree of proliferation changed with CPNE7 level. The wound healing assay showed that CPNE7 accelerated the healing rate of DLD1 cells (**Figure 2F**). In the colony formation assay, the CPNE7-OE cells had increased invasion compared with the control cell line, while the CPNE7-KD cell line had the opposite tendency (**Figure 2G**). Transwell assay showed that CPNE7-OE enhanced invasion and migration ability in DLD1 cells, while the CPNE7-KD inhibited those capacities in HCT116 (**Figures 2H, I**).

### 3.3 CPNE7 accelerates cell-derived xenograft growth

**Figure 3:**
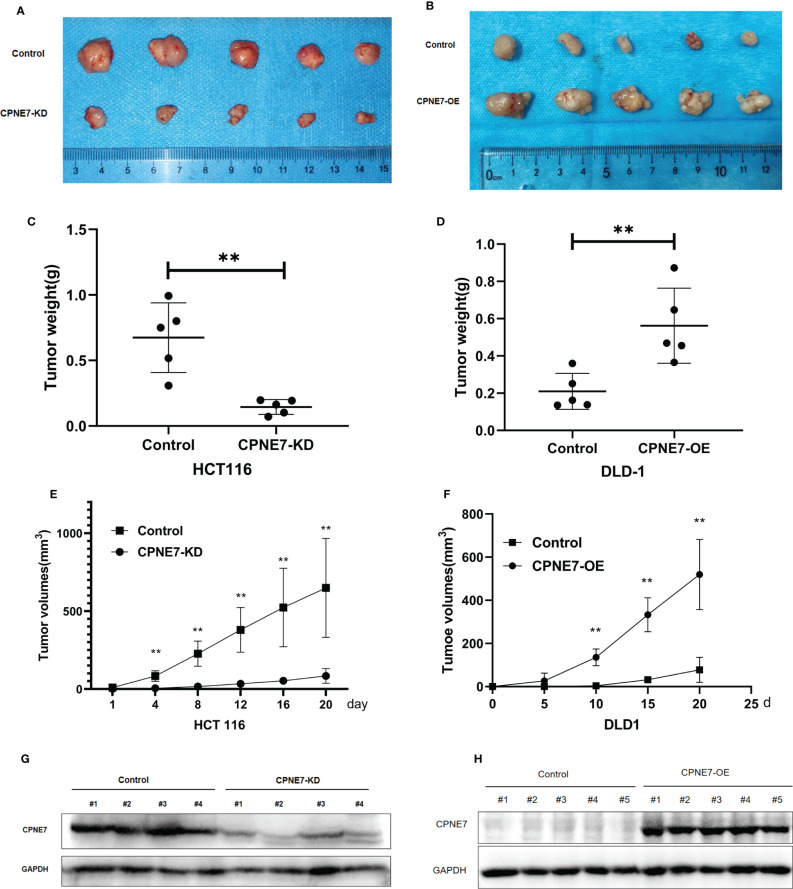
**(A)** Tumors resulting from inoculation of CPNE7-KD and control HCT116 cells in NOD-SCID mice. **(B)** Tumors resulting from inoculation of CPNE7-OE and control DLD1 cells in NOD-SCID mice. **(C)** Weight of tumors in CPNE7-KD and control groups 20 days post inoculation. **(D)** Weight of tumors in CPNE7-OE and control groups 20 days post inoculation. **(E)** Volume changes of subcutaneous tumors in CPNE7-KD and control groups. **(F)** Volume changes of subcutaneous tumors in CPNE7-OE and control groups. **(G)** WB analysis of protein expression in tumor cells collected from CPNE7-KD and control groups. **(H)** WB analysis of protein expression in tumor cells collected from CPNE7-OE and control groups. **p<0.01.

To confirm that CPNE7 regulates CRC growth *in vivo*, 20 5-week-old NOD-SCID mice were prepared for cell-derived xenograft. We subcutaneously injected CPNE7-OE cells, CPNE7-KD cells, and control cells in the mouse groups. As shown in **Figures 3A, C, E**, CPNE7-KD cells grew slowly and remained small compared with controls. **Figures 3B, D, F** show that CPNE7-OE cells rapidly grew large tumors compared with controls. The Western Blot in **Figures 3G, H** show the low expression of CPNE7-KD and high expression of CPNE7-OE, respectively. Thus, CPNE7 overexpression can promote CRC tumor proliferation *in vivo*.

### 3.4 Bioinformatic analysis based on transcriptome sequencing

**Figure 4:**
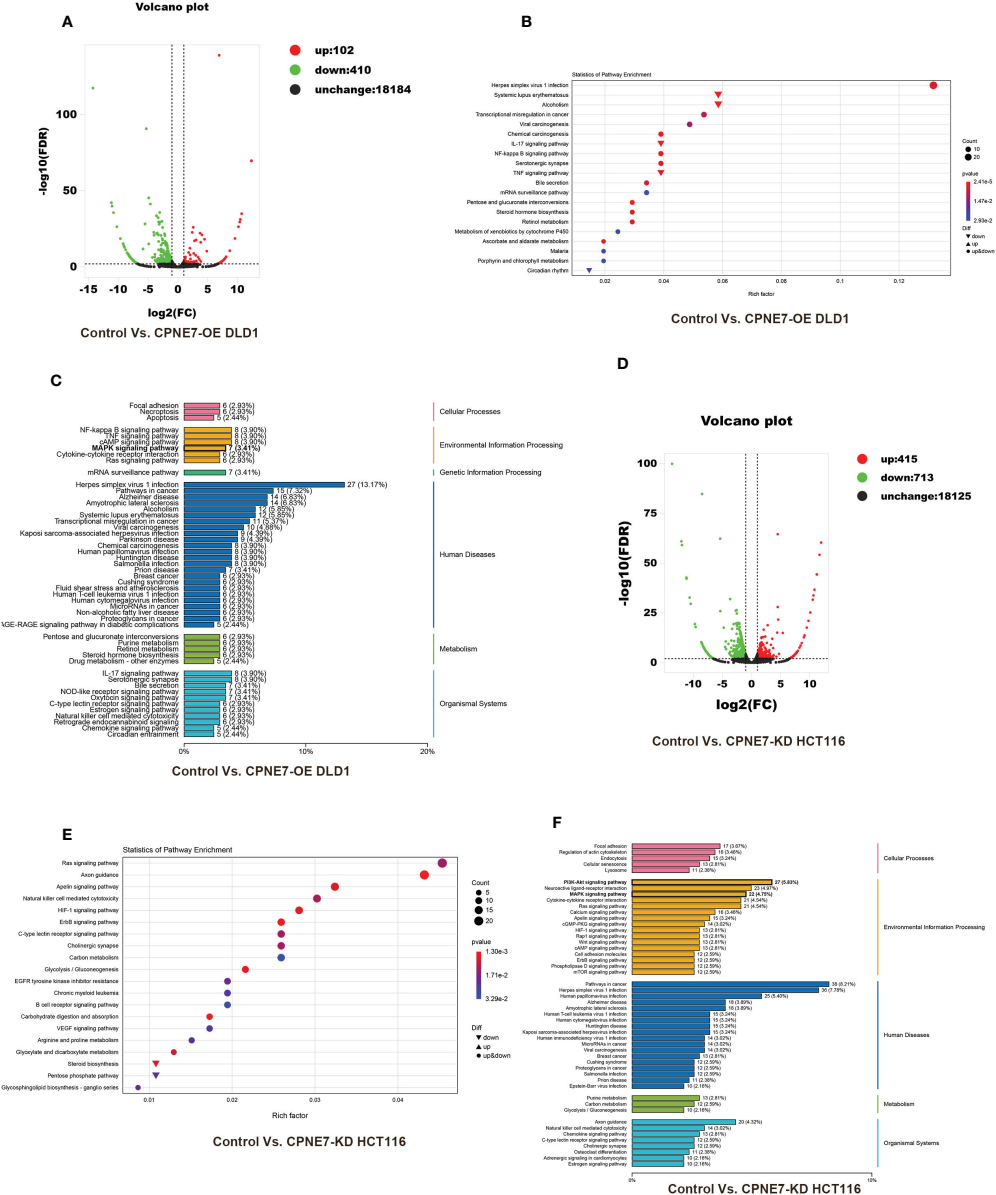
Bioinformatic analysis of DEGs between CPNE7-OE and control cells. **(A)** Volcano plot of CPNE7-OE vs. control DLD1 cells. **(B)** Transcriptomic statistics of pathway enrichment of CPNE7-OE vs. control DLD1 cells. **(C)** Transcriptomic KEGG classification analysis of CPNE7-OE vs. control DLD1 cells. **(D–F)** Bioinformatic analysis of DEGs between CPNE7-KD and control HCT116 cells. **(D)** Volcano plot of CPNE7-KD vs. control HCT116 cells. **(E)** Transcriptomic statistics of pathway enrichment of CPNE7-KD vs. control HCT116 cells. **(F)** Transcriptomic KEGG classification analysis of CPNE7-KD vs. control HCT116 cells.

To further demonstrate the mechanism of the above phenomena, we employed transcriptome sequencing analysis and Co-IP with protein liquid mass spectrometry. **Figure 4** shows the results of transcriptome sequencing of the extracted RNA of CPNE7-OE and CPNE7-KD cell lines and their control cells. Volcano plots show that high and low expression of CPNE7 induces a change in expression of other genes. In the DLD1 cell line with CPNE7-OE, 102 genes were upregulated and 410 were downregulated (**Figure 4A**). Meanwhile, in the HCT116 cell line with CPNE7-KD, 415 genes were upregulated and 713 were downregulated (**Figure 4D**). **Figure 4B** plots DEG pathways involved in various conditions including viral carcinogenesis, chemical carcinogenesis, Herpes simplex virus 1 infection, transcriptional mis-regulation in cancer, and alcoholism, compared with the control group (**Figure 4B**). With CPNE7-KD HCT116 cells, major DEG signaling pathways include RAS, HIF-1, Apelin, ErbB, and C-type lectin receptor (**Figure 4E**). Kyoto Encyclopedia of Genes and Genomes classification analysis of transcriptomes indicated that DEGs were involved in MAPK and PI3K-Akt signaling pathways (**Figures 4C, F**).

### 3.5 CPNE7 regulates Akt and ERK phosphorylation by interaction with pyruvate kinase

**Figure 5:**
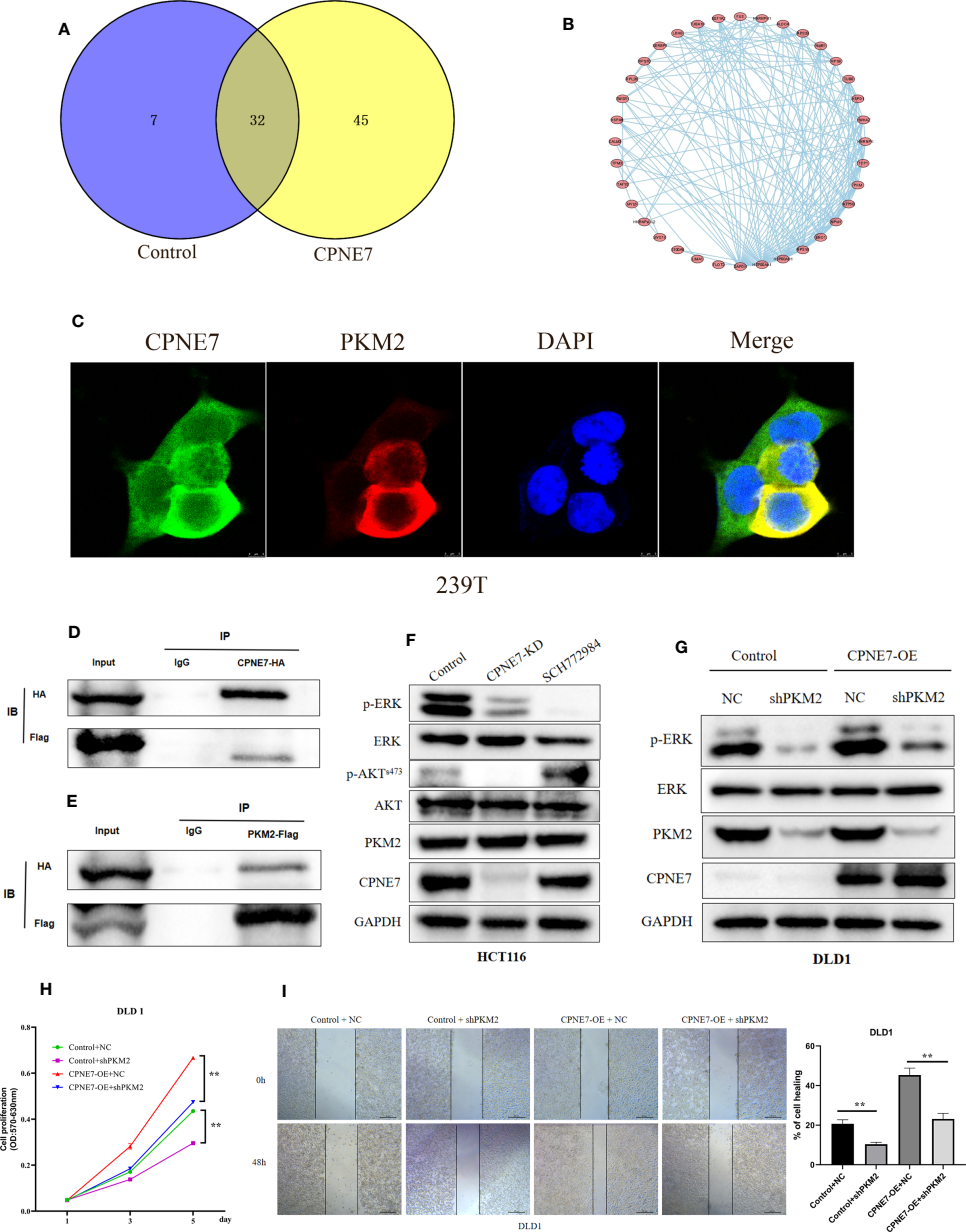
**(A)** The Venn plot between the CPNE7 and Control **(B)** The PPI network on the 45 proteins which only were detected in CPNE7-OE cell. **(C)** Immunofluorescence colocalization in 293T cell. **(D, E)** CPNE7 co-IP with PKM2 and PKM2 co-IP with CPNE7 in HEK293 cells. **(F)** WB analysis of the correlation of CPNE7 depletion in HCT116 with AKT/ERK signaling pathway. **(G)** Comparison of ERK phosphorylation differences between negative control (NC) with PKM depletion (shPKM2) in control and CPNE7-OE groups. **(H)** The MTT assay in Control, shPKM2, CPNE7-OE, and CPNE7-OE-shPKM2 cells. **(I)** Wound healing assay and the statistical result of healing rate in Control, shPKM2, CPNE7-OE, and CPNE7-OE-shPKM2 cells. **p<0.01.

We explored the underlying interactional protein by proteomic analysis using co-IP. Venn plot explored the different interactional protein in CPNE7 comparing with control (**Figure 5A**). We showed the protein interactional network on the 45 proteins which only were detected in CPNE7-OE cell using STRING and Cytoscape software (**Figure 5B**). We identified PKM2 as one of the multitudinous proteins in the Co-IP proteomic. Therefore, using the immunofluorescence colocalization assay, it proved the CPNE7 and PKM2 were co-expressing in 293T cell cytoplasm (**Figure 5C**). After co-transfecting the CPNE7-OE lentivirus and PKM2-OE lentivirus into HEK293 cells, Co-IP confirmed that exogenous CPNE7 pulled down PKM2, and vice versa (**Figures 5D, E**). PKM2, a protein kinase interacting with SAICAR, phosphorylates ERK1/2 in the MAPK signal pathway (28, 29). Interestingly, in the former transcriptome analysis, the MAPK pathway was one of the major enrichment pathways. Therefore, we hypothesized that CPNE7 regulates the MAPK pathway through interacting with PKM2. In the MAPK pathway, the level of phosphor ERK reflects the activation of a whole signaling pathway. In CPNE7-KD cells, the AKT^s473^ and ERK phosphorylation was reduced (**Figure 5F**). Moreover, when CPNE-OE cells temporarily knocked down PKM2, the phosphor ERK was dramatically decreased (**Figure 5G**). To verify the PKM2 was involved in the CPNE7-induced proliferation, we preformed the MTT assay in Control, shPKM2, CPNE7-OE, and CPNE7-OE-shPKM2 cells. Comparing with the CPNE7-OE, the rate of CPNE7-OE-shPKM2 cells proliferation is low. Moreover, the rate of shPKM2 cell proliferation is lower than the Control cells (**Figure 5H**). Meanwhile, wound healing assay also showed that the healing ability of CPNE7-OE DLD1 cells was weaken by koncking PKM2 level down (**Figure 5I**). Those results meant the PKM2 is a key protein involved into CPNE7-induced proliferation. In conclusion, these results indicate that CPNE7 activated the MAPK pathway *via* binding PKM2 and phosphorylating ERK.

## 4 Discussion

During CRC tumor development, multiple oncogenes increase expression and the stimulative signaling pathway is activated aberrantly. Normal cell proliferation is limited by regulating factors, but nevertheless, cancer cells proliferate out of control through excessive activation of the signaling pathway. The mechanism underlying cell proliferation and invasion in CRC tumors urgently needs to be explicated.

Our research verified that CPNE7 plays a key role in CRC progression, especially in tumor proliferation and invasion. Analyzing the clinicopathological characteristics, we observed that CPNE7 upregulates in CRC and is related to tumor extension. To further explore the oncogenesis of CPNE7, we employed cell function assays and animal models. The results demonstrated that CPNE7 contributes to CRC cell proliferation, motility, and invasion *in vivo* and *in vitro*. Using transcription sequencing analysis, we explored the pathways of DEGs and found that MAPK is a major enrichment pathway. Analysis of the mechanism by which CPNE7 accelerates cancer cell proliferation uncovered that CPNE7 could promote AKT and ERK phosphorylation. Meanwhile, interaction between CPNE7 and PKM2 was shown by Co-IP assay, and the low expression of PKM2 inhibited the contribution of CPNE7 to ERK phosphorylation.

CPNEs have been reported to influence many diseases, with various CPNEs having different functions. For example, CPNE6 could regulate spontaneous neurotransmission as a Ca^2+^-dependent suppressor of spontaneous release (30). CPNE3 could promote cell motility by interacting with epithelial membrane protein 1 in prostate cancer cells (12). The C2 structural domain in CPNEs plays a vital role in ERK phosphorylation (10). CPNE7 is one member of the CPNEs family; however, the CPNE7 has not been widely studied, particularly in relation to CRC. Based on bioinformatical analysis of transcriptome sequencing and Co-IP, our research demonstrated that CPNE7 acts as an oncogene, possibly by binding with PKM2 in CRC cells to activate the MAPK pathway.

In summary, our study showed that CPNE7 is over-expressed in CRC tissues and promotes cancer cell proliferation. Furthermore, CPNE7 contributes to AKT and ERK phosphorylation. Interestingly, the interaction between CPNE7 and PKM2 may be what results in the phosphorylation of ERK, thereby accelerating colorectal cancer cell proliferation.

Although we found CPNE7 could phosphorylate ERK in the MAPK signaling pathway, the specific mechanism of MAPK pathway activation remains unknown. If we can find the precise site where CPNE7 phosphorylates in the MAPK pathway, perhaps CPNE7 could be applied to clinical practice as a biomarker or anti-cancer target.

## Data availability statement

The original contributions presented in the study are included in the article/supplementary material, further inquiries can be directed to the corresponding authors.

## Ethics statement

The animal study was reviewed and approved by Laboratory Animal Welfare Ethics Committee, Central South University. Written informed consent was obtained from the individual(s) for the publication of any potentially identifiable images or data included in this article.

## Author contributions

TY contributed to conception and design of the study and wrote the first draft of the manuscript. CH revised it critically for important intellectual content and performed the statistical analysis. CL and QH wrote sections of the manuscript. ZC and WY agreed to be accountable for all aspects of the work in ensuring that questions related to the accuracy and provide approval for publication of the content. All authors contributed to the article and approved the submitted version.
